# SharDif: Sharing and Differential Learning for Image Fusion

**DOI:** 10.3390/e26010057

**Published:** 2024-01-09

**Authors:** Lei Liang, Zhisheng Gao

**Affiliations:** 1 College of Aerospace Engineering, Nanjing University of Aeronautics and Astronautics, Nanjing 210016, China; skywork@163.com; 2 Low Speed Aerodynamics Institute, China Aerodynamics Research and Development Center, Mianyang 621000, China; 3 School of Computer and Software Engineering, Xihua University, Chengdu 610039, China

**Keywords:** image fusion, shared feature, differential feature, multi-level semantic feature

## Abstract

Image fusion is the generation of an informative image that contains complementary information from the original sensor images, such as texture details and attentional targets. Existing methods have designed a variety of feature extraction algorithms and fusion strategies to achieve image fusion. However, these methods ignore the extraction of common features in the original multi-source images. The point of view proposed in this paper is that image fusion is to retain, as much as possible, the useful shared features and complementary differential features of the original multi-source images. Shared and differential learning methods for infrared and visible light image fusion are proposed. An encoder with shared weights is used to extract shared common features contained in infrared and visible light images, and the other two encoder blocks are used to extract differential features of infrared images and visible light images, respectively. Effective learning of shared and differential features is achieved through weight sharing and loss functions. Then, the fusion of shared features and differential features is achieved via a weighted fusion strategy based on an entropy-weighted attention mechanism. The experimental results demonstrate the effectiveness of the proposed model with its algorithm. Compared with the-state-of-the-art methods, the significant advantage of the proposed method is that it retains the structural information of the original image and has better fusion accuracy and visual perception effect.

## 1. Introduction

In many monitoring fields, it is difficult for a single sensor to capture enough information required to meet the monitoring tasks [1]. Sensors in different wavebands (for example, infrared and visible light) have obvious advantages in monitoring the same scene. However, on the one hand, multiple sensors bring data storage challenges, and on the other hand, the image information contained by a single sensor is flawed. Taking infrared and visible light images as an example, infrared sensors reflect the radiation characteristics of foreground targets via thermal radiation imaging, but infrared images often lack structural and texture information. The visible light sensor describes the background details of the scene via light reflection, but it is greatly affected by changes in lighting and weather conditions [2]. Therefore, image fusion has become a popular research field [3]. Image fusion is to fuse the input images into one image. At the same time, the fused image contains all the information of the input images and can even generate more significant information.

According to different application scenarios, image fusion is mainly divided into multi-focus [4], multi-spectral [5], and medical images [6]. The most studied multi-spectral image fusion is the fusion of infrared and visible light images, which also includes the fusion of hyperspectral images in the field of remote sensing. The fused multi-focus image can clearly image the background and foreground at the same time. The fused image of the multi-spectral image contains imaging information of multiple spectra. The fused image of magnetic resonance imaging (MRI) and computed tomography (CT) can clearly see the soft tissue and the bones at the same time.

The two core tasks of image fusion are feature extraction and feature fusion strategies. The original images are transformed into the feature domain, where the fusion rules are designed for features fusing, and then the fused features are reconstructed back to the original pixel space to obtain the fused image. For feature extraction tasks, existing pioneer image fusion works are divided into two major categories: methods of artificially designed transformations and methods based on feature representation learning. Feature fusion strategies are also divided into two types: manual design and global optimization learning.

Artificially constructed feature extraction methods and feature fusion rules at all levels are the most intensively studied areas of image fusion. Since such methods do not require training and are completely unsupervised methods, they have good versatility. The main feature transformation methods include discrete wavelet transform (DWT) [7], shearlet transform [8], nonsubsampled contourlet transform [9], low-rank representation (LRR) [10], and bilateral filter [11]. The manually designed fusion rules mainly include maximum value, average value, and nuclear norm. The usual approach is base parts adopt the average fusion rule, and detail parts adopt the maximum fusion rule [12]. In the representation learning domain, the typical methods are based on sparse representation (SP) [8,13,14]. SP learns a single-layer common over-complete dictionary from the input images, then performs sparse representation of the input images respectively; fuses the sparse coefficients; and reconstructs the fused image. Deep learning has better representation learning capabilities than SP, and it has become a popular research point in the field of image fusion [2,4,15,16,17,18]. Methods based on deep learning first train a common encoder and decoder using a large number of images; use the encoder to extract features of the input images respectively; use a fusion rule to fuse these feature maps; and finally, the decoder is used to reconstruct the fusion image [15,19].

For the fusion of representation features, manually designed fusion rules lack interpretability. Some research works try to learn fusion rules by defining loss functions. Global optimization methods such as particle swarm [20], Grasshopper [21], and membrane computing [22] are used for fusion rule learning. Another class of methods learns the fused decision map via deep learning [23,24].

Therefore, the core of image fusion is to transform the image from the original pixel space to a feature representation space that is easy to fuse. After fusion is achieved in the new feature space, the fused image is obtained via inverse transformation. Judging from the current research trends in academia, it is difficult to develop a new method in the traditional field of multi-scale transformation, and methods based on deep learning are the current research hotspots. The current general idea based on deep learning methods is to implement feature extraction via an encoder. After fusing the features, image fusion is achieved via a decoder. The core problem of these methods is weak interpretability and lack of criteria for judging the quality of extracted features. To address this problem, combined with the goal of merging complementary information in image fusion, we propose a new image fusion method based on shared and difference learning. The key motivation is to extract shared common features and differentiated individual features from input multi-source images. The fusion of complementary information is achieved via maximum operation. The fused image is then reconstructed via a densely connected encoder.

The major contributions of this paper include the following aspects:

(1) A novel shared and differential feature learning image fusion method is proposed. In this model, a channel with shared weights realizes the extraction of shared features from multi-source images. Separate channels are used to extract personalized differential features of the input images.
(2) In the fusion stage, a weighted fusion strategy based on an entropy-weighted attention mechanism is proposed and used to fuse the learned shared and differential features, which makes the fused image have richer texture information.
(3) A light enhanced dense module (EDB) is proposed to extract low-level semantic features, and BiFormer is used to extract high-level semantic features.
(4) Experiments were conducted on three typical datasets. Compared with the state-of-the-art fusion method, our proposed method has better performance, with obvious advantages in information fidelity and visual saliency. It shows that our method is obviously competitive.

## 2. Related Work

This paper takes infrared and visible light image fusion as an application case, focusing on complementary feature extraction of input images. At the same time, the motivation of this paper is to use deep learning to extract interpretable features from input multi-source images. Therefore, in this section, the research progress of feature extraction methods is introduced. Some methods are also used for comparative analysis in the experiments.

### 2.1. Model-Based Feature Extraction Method

Performing pixel-level transformation on the input source images and extracting multi-scale features of the original images are hot spots in early research. The image features extracted by such methods have very nice interpretability. They decompose the input images into low-frequency (base) parts and high-frequency (detail) parts. The high-frequency part reflects the basic semantic information of the scene, and the high-frequency part reflects the target information in the background. The nonsubsampled contourlet transform (NSCT) [9] is a pioneer work that is used for image fusion. To combine the advantages of multi-scale and deep learning, Wang et al. [25] proposed an image fusion method based on a convolutional neural network and NSCT. MDLatLRR [26] is a baseline method in this field, which first performs a multi-level low-rank sparse decomposition of the input images, and then fuses the base and detail parts separately. Li et al. [27] performed norm optimization on the fused images of MDLatLRR to obtain more significant fused images. Gaussian difference is used for image fusion, which is simple, efficient, and versatile [28].

### 2.2. Generative-Based Methods

GAN-based methods attempt to use generative neural network models to generate fused images conditioned on inputting multi-source images [29]. DDcGAN [30] drives the deep neural network to learn complementary features to reconstruct the fused image based on a defined loss function. GAN-FM [31] introduces a full-scale skip-connected generator and Markovian discriminators, and Fusion-UDCGAN [32] adopts a U-Type densely connected generation adversarial network. AT-GAN [33] proposes a generative adversarial network with intensity attention modules and semantic transition modules. This type of method can obtain additional image enhancement effects based on the definition of the loss function.

### 2.3. Task-Driven Approach

Fusion to improve target segmentation accuracy in low-light environments is one of the current research hotspots. This type of method hopes that the fused image will have higher brightness and more prominent target contours. SCFusion [24] achieves target saliency enhancement via a mask of the target area. SGFusion [34] achieves saliency guidance in the fusion process through multi-tasking of target segmentation. TIM [35] proposes a constrained strategy to incorporate information from downstream tasks to guide the unsupervised learning process of image fusion. SOSMaskFuse [36] also uses a target segmentation mask to achieve target enhancement. PIAFusion [37] realizes image fusion under low light conditions. This type of method generally has an enhanced effect on the original multi-source images, and the fused image has better gradient and visual saliency. But, the consistency with the original images is poor.

### 2.4. Autoencoder-Based Methods

The fusion method based on the autoencoder and decoder believes that neurons have a stronger amplitude response to salient areas. Fu et al. [38] proposed a dual branch network encoder to learn richer features. DeepFuse [39] performs feature extraction on multiple channels and is used for multi-exposure image fusion. DenseFuse [40] introduces a dense block in the encoder to extract multi-scale features. FusionDN [41] also uses a densely connected network and defines a multi-task loss function. NestFuse [42] introduces a nest connection architecture and also introduces a spatial attention mechanism to enhance the salient features. RFNNest [43] proposes a residual fusion network and can better retain detailed features. PSFusion [44] presents a practical infrared and visible image fusion network based on progressive semantic injection and scene fidelity constraints and the fusion images have good visual appeal. CDDFuse [45] is inspired by multi-scale decomposition and uses neural networks to decompose images into basic parts and detailed parts. The fused image is reconstructed after fusing the two parts respectively. This method requires two stages of training.

## 3. Proposed Method

### 3.1. The Neural Network Model

**Figure 1:**
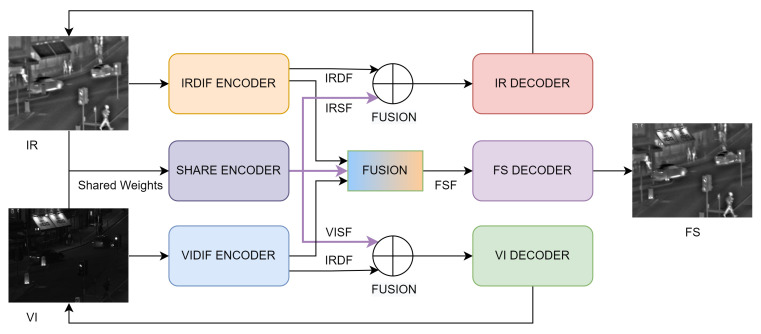
The overall framework for ShareDif.

Our proposed shared and differential feature learning image fusion model consists of encoders, a fusion layer, and decoders, as shown in Figure 1. The encoder part contains four channels, and the share feature encoders (SHE) share weights and thus have exactly the same parameters. The other two encoders extract the differential features of infrared and visible light respectively. In the fusion part, the fusion of shared features and differential features is achieved, and then the fused image is reconstructed via the fusion encoder. This is a multi-task architecture that constrains the encoder to extract the correct features via the reconstruction of the input image. The top branch implements auto-encoding reconstruction of infrared images, expressed as

(1) $$\overset{\wedge }{IR}=IRD\left[IRDIFE(IR)⊕SHE(IR)\right],$$

where IR represents the reconstructed image, IR represents the input original images, IRD(·) represents the decoder of the infrared image, IRDIFE(·) represents the difference feature encoder of the infrared image, SHE(·) is the shared feature encoder, and ⊕ is a point-wise addition operator, which achieves the fusion of shared features and differential features of a single modality image. The middle branch generates the fused image, expressed as

(2) $$FS=FSD\left\{MAX\left[IRDIFE(IR),VIDIFE(VI)\right]⊕MAX\left[SHE(IR),SHE(VI)\right]\right\},$$

where FS is the final fused image, FSD(·) is the encoder of the fused image, VIDIFFE(·) is the visible light encoder, and vi represents the input visible light image. MAX(·,·) represents point-wise maximum fusion rules, which intuitively reflect the fusion of complementary characteristics of the input multi-source images. The bottom branch implements the decomposition and reconstruction task of visible light images, expressed as

(3) $$\overset{\wedge }{VI}=VID\left[VIDIFE(\mathrm{VI})⊕SHE(\mathrm{VI})\right],$$

where VID(·) is the visible light image decoder.

#### 3.1.1. Branch Structure

**Figure 2:**
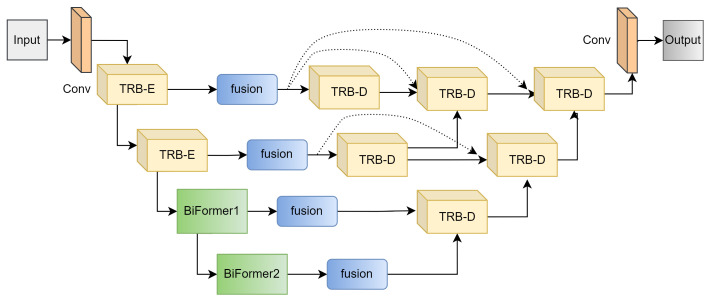
The structure of the encoder and decoder branches.

In order to better extract the shared and differential features of multi-source input images, we carefully designed the autoencoder branch structure based on existing research work, as shown in Figure 2. The encoder part is a multi-scale feature extraction module containing five layers. The first layer is a 1∗1 convolutional layer. Next are two lightweight external attention rest block encoders (TRB-E). The feature map is downsampled by 2× before the second TRB-E. The last two layers consist of two BiFormer [46] modules. Ordinary convolution is responsible for extracting the low-level features of the input images, mainly the details and texture information of the image. BiFormer is responsible for extracting high-level semantic features and making up for the lack of convolution’s ability to extract global features. The decoder consists of multiple TRB-D blocks, skip links, and a normal convolution. The decoder used can better fuse the low-level detail information and high-level semantic information of the images. The obtained fused image is semantically consistent with the input images and has better texture detail information.

#### 3.1.2. TRB Block

**Figure 3:**
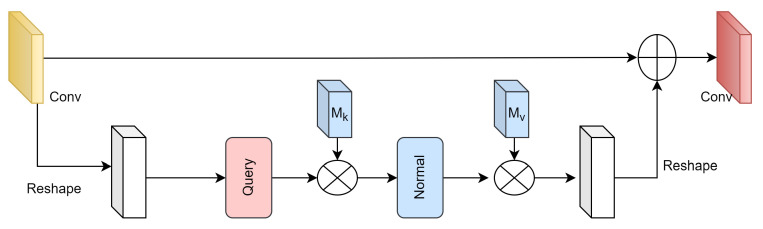
The structure of the TRB block.

In order to better extract the features contained in the image, we developed the external attention rest block (TRB) based on a lightweight resnet block and external attention, as shown in Figure 3. TRB blocks contain two convolutions (3∗3) and an external attention module. Among them, the external attention module is used to enhance the global information acquisition capability of rest blocks. Given an input feature map $F\in R^{N\times d}$, *N* is the number of pixels in the image, and *d* is the feature dimension. $M_{k}$ and $M_{v}$ are two memory units. External attention can be expressed as

(4) $$A={\left(\alpha \right)}_{i,j}=Norm\left(FM_{k}^{T}\right),$$

(5) $$F_{out}=AM_{v},$$

where ${\left(\alpha \right)}_{i,j}$ is the similarity between the i-th element and the j-th row of matrix *M*, and *M* is a learnable parameter independent of the input, which will be used as the memory of the entire training set. *A* is the attention map derived from the learned prior knowledge.

#### 3.1.3. Biformer Block

**Figure 4:**
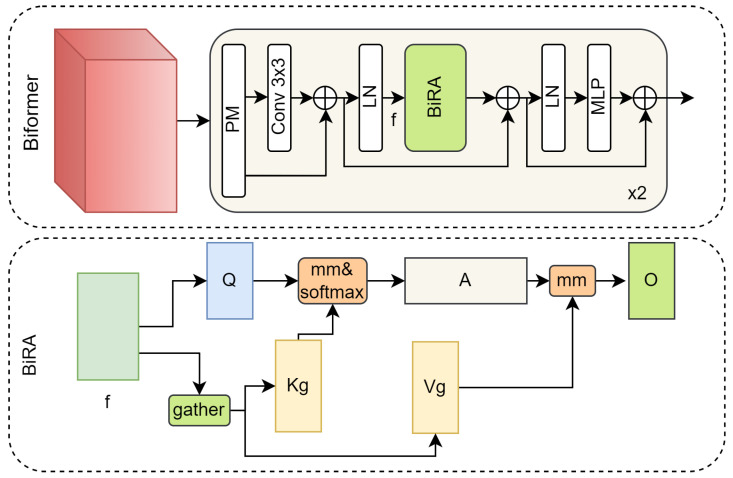
The structure of the Biformer block.

The transformer has advantages in capturing long-distance contextual dependencies and high-level semantic features. Biformer demonstrates competitiveness in a variety of visual tasks. We introduced a simplified Biformer module in the encoder, as shown in Figure 4. The input feature maps are first subjected to patch merging (PM), and then the relative position information is implicitly encoded via a 3 × 3 convolution. Then, layer normalization (LN), Bi-Level Routing Attention (BiRA), and a Multi-Layer Perceptron (MLP) are applied in sequence.

BiRA is the core of Biformer. It proposes a novel dynamic sparse attention via bi-level routing to achieve more flexible computing allocation and content awareness, as shown in Figure 4. For the input feature map *f*, the calculation process of BiRA is expressed as [46]

(6) $$Q=fW^{q},$$

(7) $$Kg=gather\left(K,I_{r}\right),Vg=gather\left(V,I_{r}\right),$$

(8) O=Attention(Q,Kg,Vg)+LCE(V),

where $W^{q}$ is a projection weight for the query, $I_{r}$ is the routing index matrix, and LCE(·) is the local context enhancement term [47].

### 3.2. Fusion Strategy Based on Entropy-Weighted Attention Mechanism

**Figure 5:**
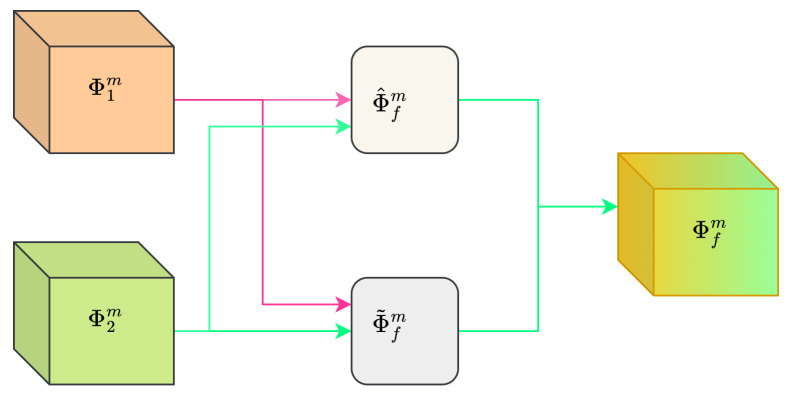
The structure of the fusion block.

After the complementary features of the source image are extracted by the encoder, a strategy is needed to fuse these features. We propose a new fusion method, which is an improvement of the attention fusion [5], which is called fusion strategy based on an entropy-weighted attention mechanism, as show in Figure 5. We apply the objective metric entropy to measure the amount of information in each source feature map on the basis of information theory [4,41,48]. The entropy of a feature map is defined as

(9) $$EN\left(\phi \right)=-\sum_{l=1}^{L-1}p_{l}log_{2}p_{l},$$

where *L* is the number of gray levels, and $p_{l}$ is the probability of the corresponding level. In the fusion layer, entropy-weighted spatial attention and channel attention mechanisms are used, and $l_{1}$-norm and softmax operations are adopted in the spatial attention mechanism. The entropy-weighted spatial attention mechanism can be expressed as

(10) $$\begin{array}{c} {\hat{\Phi }}_{f}^{m}\left(x,y\right)=\sum_{k=1}^{K}\beta_{k}^{m}\left(x,y\right)\Phi_{k}^{m}\left(x,y\right), \end{array}$$

(11) $$\begin{array}{c} {\hat{\Phi }}_{k}^{m}\left(x,y\right)=\beta_{k}^{m}\left(x,y\right)\Phi_{k}^{m}\left(x,y\right), \end{array}$$

(12) $$\begin{array}{c} \beta_{k}^{m}\left(x,y\right)=\lambda_{1}\times \frac{∥\Phi_{k}^{m}{\left(x,y\right)∥}_{1}}{\sum_{i=1}^{K}{∥\Phi_{i}^{m}\left(x,y\right)∥}_{1}}+\left(1-\lambda_{1}\right)\times \frac{EN(\Phi_{k}^{m})}{\sum_{i=1}^{K}EN\left(\Phi_{i}^{m}\right)}, \end{array}$$

where ∥·∥ represents $l_{1-norm}$; k∈{1,...,K}, *K* = 2, (x,y) represents the corresponding position in the multi-scale deep feature and weighted graph, respectively; and ${\hat{\Phi }}_{k}^{m}$ represents $\beta_{k}^{m}$-weighted deep features. Global pooling and softmax operations are used in the channel attention mechanism. Further, three types of global pooling are used: average pooling, max pooling, and kernel pooling. The entropy-weighted channel attention mechanism can be expressed as

(13) $$\begin{array}{c} {\tilde{\Phi }}_{f}^{m}\left(x,y\right)=\sum_{i=1}^{K}\alpha_{i}^{m}\left(n\right)\Phi_{i}^{m}\left(n\right), \end{array}$$

(14) $$\begin{array}{c} \alpha_{k}^{m}\left(n\right)=\lambda_{1}\times \frac{{\bar{\alpha }}_{k}^{m}\left(n\right)}{\sum_{i=1}^{K}{\bar{\alpha }}_{i}^{m}\left(n\right)}+\left(1-\lambda_{1}\right)\times \frac{EN(\Phi_{k}^{m})}{\sum_{i=1}^{K}EN\left(\Phi_{i}^{m}\right)}, \end{array}$$

(15) $$\begin{array}{c} {\bar{\alpha }}_{k}^{m}\left(n\right)=P\left(\Phi_{k}^{m}\left(n\right)\right), \end{array}$$

where n∈{1,2}, *n* is the channel number corresponding to the feature $\Phi_{k}^{m}$, and P(·) represents the pooling operation.

### 3.3. Loss Function

SharDifF is a multi-task network model with a total of three outputs, namely the reconstructed infrared image $\overset{\wedge }{IR}$, the reconstructed visible light image $\overset{\wedge }{VI}$, and the fused image FS. The encoder is constrained to extract reliable features via the reconstruction errors of $\overset{\wedge }{IR}$ and $\overset{\wedge }{VI}$, and the fused image can retain the structural information of the original input images via structural similarity. Through the maximum gradient similarity loss, the fused image can retain the edge texture features of the original images as much as possible. The loss function of the entire model is expressed as

(16) $$L_{total}=\lambda_{1}L_{ir}+\lambda_{2}L_{vi}+\lambda_{3}L_{fs}+\lambda_{4}L_{dif},$$

where $\lambda_{1}-\lambda_{4}$ are the weighting factors to balance the four losses. The four sub losses are described in detail below.

#### 3.3.1. Fidelity Loss

The purpose of reconstructing the input multi-source images is to ensure that the extracted features can reflect the essential information of the input images without causing mode collapse. We call it fidelity loss, which includes $L_{ir}$ and $L_{vi}$.

(17) $$L_{ir}=\frac{1}{HW}{∥IR-\overset{\wedge }{IR}∥}_{1}+\alpha \left(1-SSIM(IR,\overset{\wedge }{IR})\right),$$

(18) $$L_{vi}=\frac{1}{HW}{∥VI-\overset{\wedge }{VI}∥}_{1}+\alpha \left(1-SSIM(VI,\overset{\wedge }{VI})\right),$$

where ${∥\cdot ∥}_{1}$ is a norm operator, α is a balancing factor, and SSIM(·,·) is the calculation of the structural similarity between two images, which is defined as

(19) $$L_{SSIM}\left(x,y\right)=\frac{\left(2\mu_{x}\mu_{y}+C_{1}\right)\left(2\sigma_{xy}+C_{2}\right)}{\left(\mu_{x}^{2}+\mu_{y}^{2}+C_{1}\right)\left(\sigma_{x}^{2}+\sigma_{x}^{2}+C_{2}\right)},$$

where $\mu_{x}$ and $\mu_{y}$ are the averages of all pixels in the two source images; $\sigma_{x}$ and $\sigma_{y}$ are the variances of the pixel values of the two source images; and $C_{1}$ and $C_{2}$ are constants to ensure the stability of the function.

#### 3.3.2. Fusion Loss

The fusion loss $L_{fs}$ is used to obtain high-quality fusion images, which consists of three parts: content loss, gradient loss, and structure loss.

(20) $$L_{fs}=L_{con}+\beta_{1}L_{grad}+\beta_{2}\left(1-SSIM(IR,\overset{\wedge }{IR})\right),$$

where $\beta_{1}$ and $\beta_{2}$ are balance factors, and $L_{con}$ represents the content loss of the fused image, expressed as

(21) $$L_{con}=\frac{1}{HW}{∥fs-\max(IR,VI)∥}_{1},$$

where max(·,·) represents the pixel-wise maximum, and $L_{g}rad$ represents the gradient loss, which is expressed as

(22) $$L_{grad}=\frac{1}{HW}{∥\left|\nabla fs\right|-\max\left(\left|\nabla IR\right|,\left|\nabla VI\right|\right)∥}_{1},$$

where ∇ represents the gradient operator and $\left|\cdot \right|$ is the modulo operation. The fused image is consistent with the maximum gradients of the input multi-source images and has richer texture information.

## 4. Experiment and Result

### 4.1. Dataset

**Figure 6:**
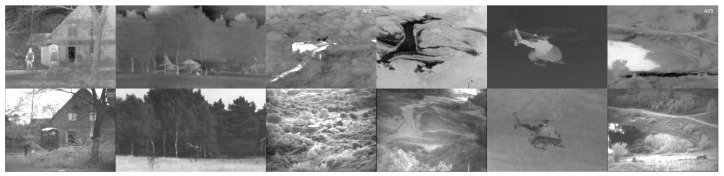
Some test samples from TNO. The top row contains infrared images, and the second row contains visible images.

**Figure 7:**
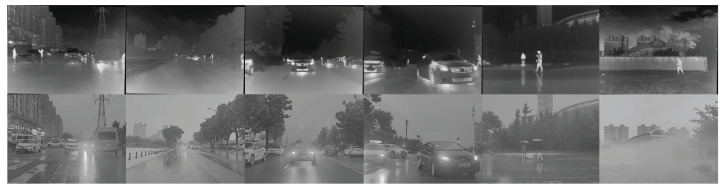
Some test samples from M3FD. The top row contains infrared images, and the second row contains visible images.

**Figure 8:**
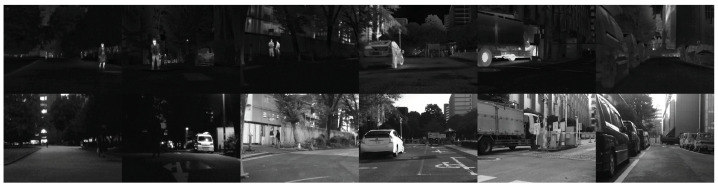
Some test samples from MSRS. The top row contains infrared images, and the second row contains visible images.

In this paper, three widely used datasets are used for experimental verification. The first dataset is TNO, whose images come from [49]. TNO contains a total of 41 pairs of infrared and visible images, of which a sample of these infrared and visible images is shown in Figure 6. The second dataset used in the paper is M3FD [50], of which this dataset contains multiple outdoor scenes, and we randomly selected 43 pairs of images from its test data for testing, as shown in Figure 7. The third dataset is MSRS [37]. This dataset contains a total of 1083 training samples. Fifty-two samples are also randomly selected from the test set to evaluate the algorithm performance, as shown in Figure 8. Since the data scale of TNO is small, the experimental models that need to be trained are uniformly trained using the MSRS training set. The trained models were used for testing on M3FD, MSRS, and TNO, respectively.

### 4.2. Methods for Comparison

In order to verify the effectiveness of the image fusion model SharDif proposed in this paper, we compare it with eight state-of-the-art image fusion methods. Optimization-based generation methods include DDcGAN [30]. Methods based on deep neural network feature representation learning include DenseFuse [40], RFNNest [43], FlFuse [51], PIAFusion [37], and PSFusion [44]. Model-based methods include GDFusion [28], and DDFM [52]. It should be noted that (1) All methods participating in the comparison use the weights and parameters given in the original papers to achieve the fusion of test data. (2) The fusion rule in denseFuse is “add”, and the fusion rule in RFNNest is “max”.

### 4.3. Evaluation Index

In order to quantitatively compare the competitiveness of various image fusion methods, nine metrics are used to compare various methods on the test set. These metrics we selected include metrics that reflect the visual consistency of images, such as mutual information (MI) [53], $Q^{AB/F}$ (petrovic metric) [54], $Q_{E}$ (piella metric) [55], and feature mutual information (FMI) [56]. Indicators reflecting information fidelity, such as IFC [57], and other comprehensive indicators, such as structural similarity index measure (SSIM) [58], peak signal-to-noise ratio (PSNR), Q0 [58], and entropy (EN) [28], are included.

### 4.4. Ablation Experiment

In this section, we first verify the effectiveness of our proposed ShareDif for image fusion on the test set MSRS. ShareDif uses a backbone network similar to RFNNest [43], which is named Model 1. On this basis, we propose the idea of shared and differential feature learning, which is named Model 2. In the feature extraction stage, we proposed the EDB module, which was named Model 3. BiFormer was introduced at the high level to extract high-level semantic features to obtain Model 4. The fusion strategy-based entropy-weighted attention mechanism is added to Model 4, and finally, the fusion model ShareDif proposed in this paper was obtained.

The experimental results of these models on MSRS are shown in Table 1. It can be seen from the results that the shared difference learning method proposed in this paper has significantly improved the performance of image fusion, especially compared with the original model, it has been improved in almost all evaluation indicators, especially MI, $Q^{abf}$, PSNR, SSIM, IFC, etc., have greatly been improved. This shows that by learning shared features and differential features, and then designing an appropriate fusion strategy, it is beneficial for the fused image to contain complementary features of the input multi-source images. The EDB module is introduced into the backbone model, especially BiFormer, which is used to extract advanced semantic features, so that the fused image and the multi-source input image have better information consistency. Several indicators such as MI, $Q^{abf}$, PSNR, and IFC have been significantly improved, indicating that the fusion image obtained by our method has better information fidelity.

### 4.5. Comparative Analysis of Experimental Results

#### 4.5.1. Visual Quantitative Comparison

**Figure 9:**
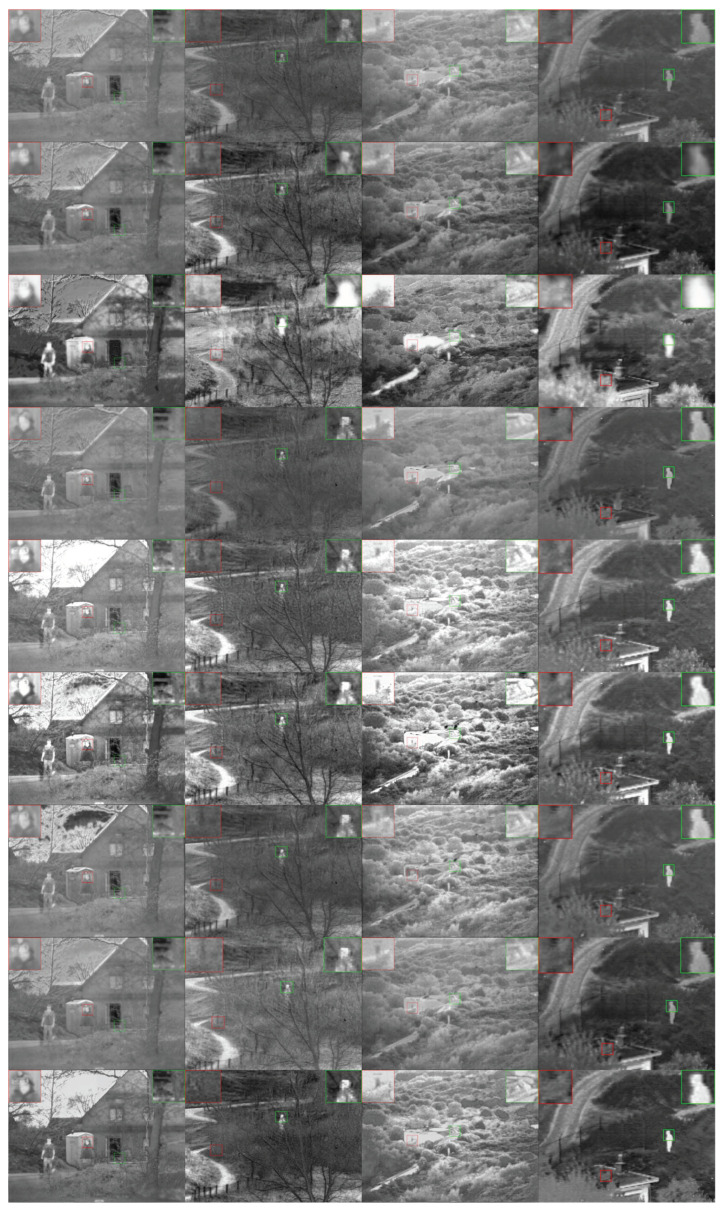
Qualitative comparison of all methods on six typical image pairs from TNO. From top to bottom: fusion results of DenseFuse [40], RFNNest [43], DDcGan [30], FLFuse [51], PIAFusion [37], PSFusion [44], GDFusion [28], DDFM [52], and our ShareDif.

TNO is a widely used dataset, its images contain many scenes, and the image resolutions also varies greatly. This dataset is small in size and has no training samples, which can reflect the generalization ability and universality of the algorithms. Six pairs of test samples were arbitrarily selected for subjective evaluation of visual effects. The fusion results of all methods are shown in Figure 9. It can be seen from the figure that the fusion results of the three methods, DenseFuse, RFN-Nest, and FIFuse are similar, and all have the disadvantages of weak image contrast and overall poor image quality. These three methods all use the COCO dataset to train the network model. They do not consider the differences in extraction features of different modal images. The advantage is that they do not require infrared and visible light image pairs for training. The disadvantage is that the fusion effect is not good. The generation-based method DDcGan can highlight the target, but the image is obviously distorted and the fused image is blurry and loses a lot of details. Both PIAFusion and PSFusion methods enhance the input multi-source images, which can improve the fusion effect under low illumination conditions. However, the fusion results of these two methods are less consistent with the source images. The advantage of the traditional method GDFusion is that it does not require training. From the fusion effect, the image consistency is poor and the target is not prominent. The target contrast of the fused image obtained using the DDFM method is low, and the overall visual saliency of the image is poor. Our method is trained on MSRS and the model can be well transferred to TNO data, indicating that ShareDif can extract the essential features of input multi-source images and complete the fusion. From the fusion effect point of view, the fusion result of ShareDif has a more obvious target. The overall image looks natural and has good consistency with the input original images.

**Figure 10:**
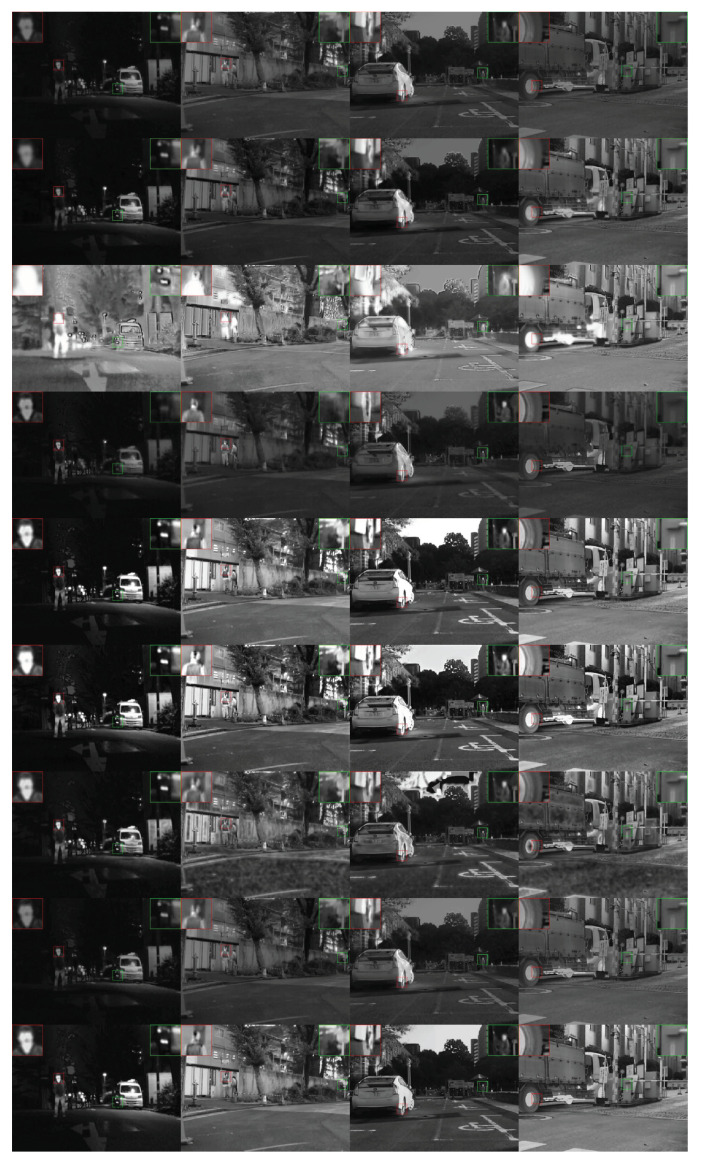
Qualitative comparison of all methods on six typical image pairs from MSRS. From top to bottom: fusion results of DenseFuse [40], RFNNest [43], DDcGan [30], FLFuse [51], PIAFusion [37], PSFusion [44], GDFusion [28], DDFM [52], and our ShareDif.

The MSRS dataset is large in scale and is mainly taken in dark environments, which is more in line with the practical application scenarios of infrared and visible light fusion. It is also a widely used dataset in recent years. Six pairs of test samples were also selected for experiments, and the fusion results of all methods are shown in Figure 10. Through observation, the targets in the fused image of FLFuse are dim and the clarity of fused images is the worst. DDcGan has a significant enhancement effect on the image, and the overall brightness is very high, but the image is also seriously distorted. The results of DenseFuse, RFN-Nest, and DDFM are relatively similar, and the brightness of cars and pedestrians in the fused image is poor. Due to edge enhancement, GDFusion’s fused image has serious noise. The fusion results of PIAFusion and PSFusion are relatively close, and some areas in the fused image have brightness oversaturation. ShareDif’s fused image has moderate brightness, and the image quality is in line with human visual imaging characteristics.

**Figure 11:**
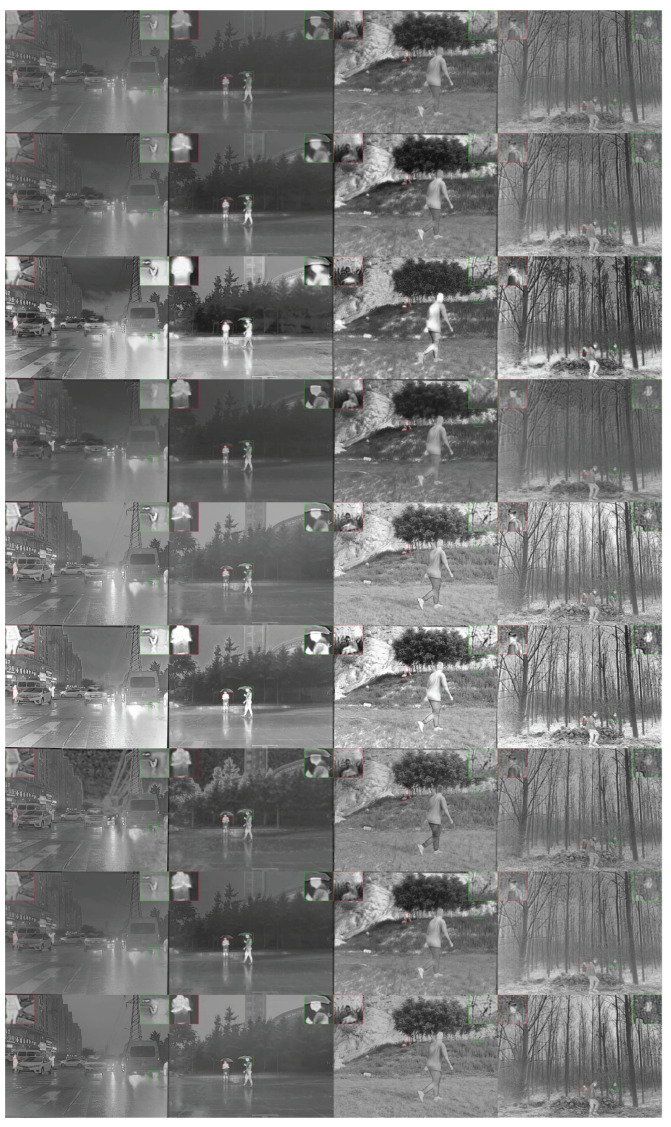
Qualitative comparison of all methods on six typical image pairs from M3FD. From top to bottom: fusion results of DenseFuse [40], RFNNest [43], DDcGan [30], FLFuse [51], PIAFusion [37], PSFusion [44], GDFusion [28], DDFM [52], and our ShareDif.

M3FD has a larger number of images and contains a wider variety of scenes. Its visible light image has high brightness and serious noise. The test results of six samples are shown in Figure 11. Consistent with the previous conclusion, the image contrast and clarity of the fusion results of DenseFuse, RFN-Nest, FLFuse, and DDFM are insufficient. DDcGan, PIAFusion, and PSFusion significantly enhance images, and some areas are severely oversaturated, which affects the visual effects of the images. The results of ShareDif have the best visual effect and the target is more prominent, especially the last column of images. The fused images conform to human visual habits.

#### 4.5.2. Quantitative Comparisons

Fusion image quality evaluation is still a problem worthy of study [18,36], and different measurement indicators reflect different characteristics of fusion images. For example, Mutual information (MI) indicates how many features are preserved in fused images. $Q^{abf}$ reflects the quality of visual information obtained from the fusion of the input images. SSIM is a widely used metric which models the loss and distortion between two images according to their similarities in light, contrast, and structure information. The larger value of EN means that there is more information contained. On the other hand, EN can be affected by noise easily. The reason why multiple different metrics are needed to quantitatively compare the fusion results is that these metrics will not give consistent results. Therefore, in this paper, we selected nine indicators to compare the algorithms. In Table 2, Table 3 and Table 4, each specific metric is the average of all images in the test set.

The objective evaluation results of TNO are shown in Table 2. The fusion results of the convolutional neural network model DenseFuse and FLFuse trained using the COCO dataset have the best PSNR and SSIM. The results of DDcGan and DDFM reflect their weak information fidelity capabilities, and the fused image is greatly different from the multi-source input image. The indicators of PSFusion, GDFusion, FLFuse, and RFN-Nest are relatively close. PIA has good reviews and ranks second. For MI and IFC, our method ShareDif has very obvious advantages, indicating that the fused image of our method can greatly maintain the original information of multi-source input images.

Table 3 shows the test results of MSRS. As can be seen from the table, the SSIM indicators of DenseFuse, FLFuse, PIAFusion, and GDFusion are relatively close, indicating that they all pay more attention to the fusion of image texture edges. The fidelity capabilities of DDcGan and DDFM are still the worst. The results of PIAFusion and PSFusion are relatively close and have better $Q^{abf}$. ShareDif has a leading position in multiple indicators, especially MI and IFC, which is much higher than the second-ranked one, and its advantages are obvious.

The evaluation results of M3FD are shown in Table 4. Three methods, including DenseFuse, RFN-Nest, and FLFuse, have the best SSIM. PIAFusion and ShareDif have clear leading advantages. ShareDif significantly outperforms the remaining methods for PSNR. The results on three different datasets can reflect the generalization performance of the algorithm. Our method ShareDif has the strongest information fidelity and has obvious advantages on all three datasets.

## 5. Conclusions

In this paper, an intuitively motivated image fusion method is proposed. Since different multi-source images contain complementary information, the goal of image fusion is to retain the complementary information of the multi-source input images in the resulting image as much as possible. We propose an image fusion model of shared and differential learning, which can achieve information extraction from multi-source input images. The shared weight branch is used to extract common information from the input images, and the other channels are used to learn the differentiated features of the input images. Based on the semantic decomposition of the input images, the complementary information of the original images is fused. Our fusion model only requires one training session to optimize the model. Extensive experiments have been performed to demonstrate the proposed method’s superiority, including texture and edge preservation, illumination adaptation, and comprehensive performance in visual effects and metrics. In the future, similar ideas can be considered for multi-focus and medical image fusion, as well as primary image processing such as denoising and super resolution.

## Figures and Tables

**Table 1:** Ablation experiment results on MSRS dataset.

Metric	EN	MI	$Q^{abf}$	$Q_{0}$	$Q_{e}$	PSNR	FMI	SSIM	IFC
Model 1	5.97	2.73	0.294	0.303	0.464	13.75	0.42	0.541	5.94
Model 2	6.35	4.34	0.637	0.461	0.467	19.65	0.461	0.714	8.90
Model 3	6.61	4.55	0.676	0.477	0.472	19.71	0.471	0.722	9.67
Model 4	6.63	4.73	0.681	0.482	0.475	20.50	0.472	0.720	10.12
ShareDif	6.65	4.71	0.683	0.491	0.482	20.57	0.475	0.721	10.17

**Table 2:** Quantitative Comparisons of all methods on TNO (The bold is best).

Metric	EN	MI	$Q^{abf}$	$Q_{0}$	$Q_{e}$	PSNR	FMI	SSIM	IFC
DenseFuse (2018)	6.35	2.21	0.36	0.47	0.28	16.00	0.43	**0.74**	5.09
RFNNest (2021)	6.42	2.67	0.32	0.29	0.29	13.14	0.44	0.58	4.29
DDcGan (2020)	**7.37**	1.84	0.36	0.36	0.16	13.04	0.41	0.59	4.42
FLFuse (2022)	6.36	2.17	0.41	**0.49**	0.28	**16.27**	0.42	**0.74**	5.60
PIAFusion (2022)	6.90	3.16	0.53	**0.49**	0.35	14.60	0.44	0.69	7.70
PSFusion (2023)	7.26	2.29	0.53	0.44	0.32	14.26	0.42	0.64	5.76
GDFusion (2023)	6.61	2.06	0.50	0.45	0.25	15.73	0.43	0.72	5.28
DDFM (2023)	6.84	1.74	0.26	0.23	0.10	14.40	0.25	0.56	2.06
ShareDif	7.08	**3.65**	**0.54**	0.48	**0.36**	14.61	**0.44**	0.70	**8.42**

**Table 3:** Quantitative Comparisons of all methods on MSRS (The bold is best).

Metric	EN	MI	$Q^{abf}$	$Q_{0}$	$Q_{e}$	PSNR	FMI	SSIM	IFC
DenseFuse (2018)	5.96	2.67	0.37	0.45	0.38	18.91	0.43	**0.74**	5.28
RFNNest (2021)	5.97	2.73	0.29	0.30	0.47	13.76	0.43	0.54	5.94
DDcGan (2020)	**7.24**	1.88	0.38	0.24	0.20	9.77	0.37	0.39	3.57
FLFuse (2022)	5.78	2.14	0.29	0.42	0.31	19.25	0.43	0.72	5.08
PIAFusion (2022)	6.64	3.99	0.67	0.48	0.45	20.42	0.46	0.71	8.31
PSFusion (2023)	6.87	2.92	0.68	0.46	0.47	18.14	0.46	0.68	7.14
GDFusion (2023)	6.36	2.31	0.62	0.44	0.38	18.35	0.43	0.71	5.70
DDFM (2023)	6.14	2.35	0.42	0.39	0.32	18.77	0.32	0.69	4.09
ShareDif	6.65	**4.71**	**0.69**	**0.49**	**0.48**	**20.57**	**0.48**	0.72	**10.17**

**Table 4:** Quantitative Comparisons of all methods on M3FD (The bold is best).

Metric	EN	MI	$Q^{abf}$	$Q_{0}$	$Q_{e}$	PSNR	FMI	SSIM	IFC
DenseFuse (2018)	6.43	2.89	0.38	0.48	0.43	16.27	0.43	0.76	6.13
RFNNest (2021)	6.44	2.45	0.40	0.46	0.35	16.76	0.38	**0.78**	5.38
DDcGan (2020)	**7.41**	2.54	0.49	0.37	0.34	13.65	0.42	0.64	5.32
FLFuse (2022)	6.51	3.20	0.27	0.46	0.33	16.59	0.41	0.75	5.50
PIAFusion (2022)	6.83	4.19	**0.64**	0.49	0.51	16.41	0.46	0.71	9.60
PSFusion (2023)	7.39	2.74	0.58	0.42	0.46	13.39	0.42	0.65	6.51
GDFusion (2023)	6.79	2.09	0.61	0.37	0.42	15.91	0.39	0.70	5.93
DDFM (2023)	6.68	2.77	0.44	0.42	0.40	16.01	0.35	0.72	5.07
ShareDif	6.76	**4.56**	**0.64**	**0.49**	**0.52**	**17.18**	**0.47**	0.73	**10.21**

## Data Availability

The data presented in this study are available on request from the corresponding author.
